# Supplementary material for: HIVprotI: an integrated web based platform for prediction and design of HIV proteins inhibitors
Source: J Cheminform. 2018 Mar 9;10:12. doi: 10.1186/s13321-018-0266-y (PMC5845081; doi:10.1186/s13321-018-0266-y)
Supplement: Supplementary file 2 — Additional file 2. Source code of HIVProtI web server. [file 13321_2018_266_MOESM2_ESM.zip › HIVprotI_Source-code/hivprotI/weka-3-6-6/doc/weka/classifiers/bayes/net/search/fixed/NaiveBayes.html]

NaiveBayes


---


|  |  |  |
| --- | --- | --- |
| **Overview** \| **Package** \| **Class** \| **Tree** \| **Deprecated** \| **Index** \| **Help** \| **Weka's home** | |  |
| **PREV CLASS**   NEXT CLASS | **FRAMES**    **NO FRAMES**     **All Classes** |  |
| SUMMARY: NESTED \| FIELD \| CONSTR \| METHOD | DETAIL: FIELD \| CONSTR \| METHOD |  |


---


## weka.classifiers.bayes.net.search.fixed Class NaiveBayes

```
java.lang.Object
  weka.classifiers.bayes.net.search.SearchAlgorithm
      weka.classifiers.bayes.net.search.fixed.NaiveBayes
```

**All Implemented Interfaces:**: java.io.Serializable, OptionHandler, RevisionHandler

---

``` public class NaiveBayes extends SearchAlgorithm ```

The NaiveBayes class generates a fixed Bayes network structure with arrows from the class variable to each of the attribute variables.


**Version:**
:   $Revision: 1.6 $

**Author:**
:   Remco Bouckaert

**See Also:**: Serialized Form

---

**Constructor Summary**

`NaiveBayes()`


| **Method Summary** | |
| --- | --- |
| `void` | `buildStructure(BayesNet bayesNet, Instances instances)`             buildStructure determines the network structure/graph of the network. |
| `java.lang.String` | `getRevision()`             Returns the revision string. |
| `java.lang.String` | `globalInfo()`             Returns a string describing this object |

**Methods inherited from class weka.classifiers.bayes.net.search.SearchAlgorithm**

`getOptions, initAsNaiveBayesTipText, listOptions, maxNrOfParentsTipText, setOptions, toString`

**Methods inherited from class java.lang.Object**

`equals, getClass, hashCode, notify, notifyAll, wait, wait, wait`

**Constructor Detail**

### NaiveBayes

```
public NaiveBayes()
```


**Method Detail**

### globalInfo

```
public java.lang.String globalInfo()
```

:   Returns a string describing this object

    :   **Returns:**: a description of the classifier suitable for displaying in the explorer/experimenter gui

---


### buildStructure

```
public void buildStructure(BayesNet bayesNet,
                           Instances instances)
                    throws java.lang.Exception
```

:   **Description copied from class: `SearchAlgorithm`**
:   buildStructure determines the network structure/graph of the network.
    The default behavior is creating a network where all nodes have the first
    node as its parent (i.e., a BayesNet that behaves like a naive Bayes classifier).
    This method can be overridden by derived classes to restrict the class
    of network structures that are acceptable.

    :   **Overrides:**: `buildStructure` in class `SearchAlgorithm`
    :   **Parameters:**: `bayesNet` -: `instances` - the instances to work with **Throws:**: `java.lang.Exception` - if something goes wrong

---


### getRevision

```
public java.lang.String getRevision()
```

:   Returns the revision string.

    :   **Specified by:**: `getRevision` in interface `RevisionHandler` **Overrides:**: `getRevision` in class `SearchAlgorithm`
    :   **Returns:**: the revision


---


|  |  |  |
| --- | --- | --- |
| **Overview** \| **Package** \| **Class** \| **Tree** \| **Deprecated** \| **Index** \| **Help** \| **Weka's home** | |  |
| **PREV CLASS**   NEXT CLASS | **FRAMES**    **NO FRAMES**     **All Classes** |  |
| SUMMARY: NESTED \| FIELD \| CONSTR \| METHOD | DETAIL: FIELD \| CONSTR \| METHOD |  |


---
